# High-throughput sequencing explores the genetic variability of endophytic bacteria in three Sichuan bamboo species (*Phyllostachys edulis*, *Bambusa rigida*, and *Pleioblastus amarus*)

**DOI:** 10.3389/fmicb.2024.1501057

**Published:** 2025-02-19

**Authors:** Kuan Yan, Xinyi Li, Yu Cai, Lina Meng, Qin Wei, Xianming Zhao, Rania M. Y. Heakel, Amr M. Atif, Mohamed A. Abd Elhamid, Salma A. Soaud, Ahmed H. El-Sappah

**Affiliations:** ^1^Faculty of Agriculture, Forestry and Food Engineering, Yibin University, Yibin, China; ^2^Sichuan Oil Cinnamon Engineering Technology Research Center, Yibin University, Yibin, China; ^3^Department of Genetics, Faculty of Agriculture, Zagazig University, Zagazig, Egypt; ^4^Department of Microbiology, Faculty of Agriculture, Zagazig University, Zagazig, Egypt

**Keywords:** bamboo, *Phyllostachys edulis*, *Bambusa rigida*, *Pleioblastus amarus*, endophytic bacteria, 16 s rDNA sequencing

## Abstract

**Introduction:**

Bamboo is a sustainable and degradable resource for sustenance, high-strength cellulose microfibers, and synthetic fiber in China. Endophytic bacteria enhance root development and ethylene levels, benefiting the host plants’ physiology.

**Methods:**

We investigated the population, diversity, and abundance of endophytic bacteria in the leaves of three bamboo species—*Phyllostachys edulis*, *Bambusa rigida*, and *Pleioblastus amarus*—using high-throughput 16S rDNA sequencing.

**Results and discussion:**

A total of 1,159 operational taxonomic units (OTUs) were obtained and further classified into 26 phyla, 64 classes, 158 orders, 270 families, 521 genera, and 811 species. The phyla with the highest abundance were Proteobacteria, Actinobacteria, and Myxococcota, and the highest genera included 1,174–901-12, *Sphingomonas*, and unclassified_f__Enterobacteriaceae. The relative richness of endophytic bacteria in the three species was in the following order: *B. rigida* > *P. amarus* > *Ph. edulis*. The PICRUSt functional richness analysis of endophytic bacteria indicated their involvement in six biological pathways: “cellular processes,” “environmental information processing,” “genetic information processing,” “human diseases,” “metabolism,” and “organic systems.” Among the 41 sub-functions, the most common were “amino acid metabolism,” “carbohydrate metabolism,” “cell motility,” “cellular signaling,” “energy metabolism,” and “membrane transport.” Our results provide precise knowledge for better managing bamboo forests and pave the way for isolating secondary metabolites and potential bioactive compounds.

## 1 Introduction

Microbial endophytes are microorganisms that live within the tissues of plants and form symbiotic relationships with their hosts (Gond et al., 2015). Anti-pathogenic bacteria, plant endophytic bacteria, abiotic stress tolerance, and physiology enhancement in host plants are all outcomes of their interactions (Hardoim et al., 2008). Plant endophytic bacteria are significant biological resources commonly employed across various industries (Donoso et al., 2017; Yan et al., 2021a). The species richness and diverse community structure of endophytes in plants are primarily determined by various factors, including plant species, seed vector endophytes, life cycle, adjacent environment, and plant tissues (Yan et al., 2022). Diverse endophytes are found in greater abundance in leaves than in any other tissue due to their susceptibility to airborne damage, stomatal openings, and transportability (Cui et al., 2017).

Generally, distinct endophytic bacterial species inhabit a variety of plant species (Liu et al., 2021). A comprehensive examination unveiled that even variations of identical plant species support a wide array of endophyte community structures and species diversity (Massimo et al., 2015). An investigation by Yuan et al. (2014) revealed a notably heterogeneous endophytic microbiome in rhizomes procured from different varieties of moso bamboo across distinct regions. A total of 129 species and 54 genera of endophytic bacteria have been identified in diverse plant taxa (White et al., 2015). *Proteus*, *Bacillus*, *Enterobacter*, and *Azospirillum* are notably abundant genera. In addition, the diversity of the endophytic bacterial community is influenced by the surrounding environment and the diversity of the endophyte host plants. Factors such as plant species, life span, location, and various plant tissues are regulated in endophytic bacterial diversity regulation. Many endophytic bacterial strains have been identified in the aerial portions of plants, specifically the leaves, except for strains specific to particular tissues. This is because leaves are more exposed to the environment and are more delicate (Cui et al., 2017).

Bambusoideae, a member of the Poaceae family, is renowned for its rapid growth, adaptability, and versatility. Its abundant species diversity spans over 70 genera and 1,200 species across the globe (Liu et al., 2017). Bamboo is a forest resource that contributes significantly to the regional economy and contemporary forestry by incorporating ecological, social, and economic benefits. The paper and pulp industries extensively use bamboo cellulose. It is a renewable primary material utilized in the construction and furniture industries. Recently, bamboo microfibers with a strength of 1.26 ± 0.21 GPa cm^−3^ g^−1^ have been extracted; these fibers are more powerful than steel and will bring about a paradigm shift in the construction sector (Li et al., 2022). Because of its adaptability to a wide range of environmental conditions and the abundance of bamboo species, it is capable of harboring a greater variety of microbial species (Shen et al., 2014; Zhou et al., 2017). The investigation of endophytic bacteria in *Phyllostachys praecox* and *Phyllostachys edulis* has extended beyond the Shunan bamboo sea (Zhang et al., 2019). Bamboo is being developed extensively as an ecological and economic forest, but most research focuses on material quality and variety propagation. For example, the growth, composition, and mechanics of *Ph. edulis* have been the subject of numerous studies (Xu et al., 2011; Wang et al., 2012). The majority of studies on *Pleioblastus amarus* focus on its medicinal and edible properties (Ying et al., 2022). *Bambusa rigida*, a native bamboo species in Sichuan, has significant timber and papermaking resource development (Wen et al., 2011; Liu et al., 2014). However, research on *B. rigida* is currently limited and primarily concerns itself with lignocellulose. Therefore, three bamboo species with entirely different genetic characteristics and uses were selected to study the diversity of endophytic bacteria, which can help in better understanding the differences in the endophytic bacterial community structure and function between them.

Endophyte research is crucial for the development of the bamboo-based industry, the maintenance of bamboo forest ecosystems, and the cultivation of bamboo forests. Numerous microorganisms continue to be uncultivable, rendering conventional cultivation methods disappointing. The utilization of next-generation sequencing techniques has enabled a comprehensive examination of the microbiome of any given species. Therefore, three representative bamboos, *Phyllostachys edulis*, *Bambusa rigida*, and *Pleioblastus amarus*, from the Shunan Bamboo Sea, Yibin, China, were selected as the research objects. The endophytic bacteria in the leaves of the three bamboo species were analyzed by molecular biotechnology, and their community structure and diversity characteristics were revealed.

## 2 Materials and methods

The methods of this study follow relevant institutional, national, and international guidelines and legislation. This study protocol also adheres to the IUCN Policy Statement on Research Involving Species at Risk of Extinction and the Convention on the Trade in Endangered Species of Wild Fauna and Flora.

### 2.1 Plant material collection

To investigate the density and diversity of endophytic bacteria leaves, the study material was collected from *Ph. edulis* (MZ), *B. rigida* (YT), and *Pl. amarus* (KZ) located in the Shunan Bamboo Sea (Yibin, Sichuan, 28°30′2″N, 105°04′7″E), China. The sample plot of each bamboo species was set up into three blocks. In each block, five bamboos with vigorous growth, far away from the edge zone, free from pests and diseases, and with an interval of about 15 m were selected. The leaves of these bamboos were collected and mixed into a sample, which was quickly maintained at 4°C. Upon arriving at the laboratory, all appendages were trimmed away, and dirt areas on the surface of leaves were thoroughly cleaned with running tap water. Leaves of each growth stage were weighed (10 g), packed in sterile bags, and stored at 4°C for future use as research material. Surface sterilization of leaves was performed via soaking in 75% ethanol for 30 s and 2% NaClO solution for 2 min with gene shaking, followed by three washings with sterile ddH_2_O. To ensure proper surface sterilization of leaves, 20 mL of ddH_2_O from the last rinse of each sample was streaked on Potato Dextrose Agar (PDA) medium in three biological replicates and incubated at 28°C under dark conditions.

### 2.2 DNA extraction

To extract DNA, 5 g of sample was weighed, chopped into small pieces, and immediately soaked in 50 mL sterile Tween-NaCl buffer [0.9% NaCl (w/v), 0.05% Tween 20 (v/v), and 2% polyvinylpolypyrrolidone (w/v)], homogenized, and sonicated at 4°C for 30 min, as described by Yan et al. (2021b). Subsequently, the solution was filtered through a three-layered sterile gauze to remove coarse particles, followed by centrifugation at 12,298 × *g* for 10 min at 4°C. The supernatant was discarded, and the residue was shifted into a new sterile tube for genomic DNA (gDNA) extraction. We employed an E.Z.N.A™ HP Plant DNA Kit (OMEGA^®^, USA) by following the manufacturer’s standard protocol to isolate bacterial genomic DNA. To avoid noise during polymerase chain reaction (PCR), impurities in bacterial genomic DNA, such as polyphenol, were removed with the help of the E.Z.N.A^™^. Soil Kit. The quality of the bacterial genomic DNA was confirmed by running 2 μL of extracted DNA of each sample on 1% agarose gel and visualized under a UV light-installed gel documentation system (iBright 1,500 imaging system, ThermoFisher, USA) (El-Sappah et al., 2017). Finally, DNA concentrations were recorded on a nanodrop spectrophotometer (NanoDrop 2000) at OD 260/280, which were 2.1–42.5 ng/μl in each sample (El-Sappah et al., 2021).

### 2.3 PCR amplification and sequencing analysis

In order to design primers to use in PCR to amplify bacterial rRNA, we used the Premier 5 tool (Wang et al., 2018). The following primer pair, 799F(5’-AACMGGATTAGATACCCKG-3′) and 1193R(5’-ACGTCATCCCCACCTTCC-3′), was used to amplify the V5–V7 regions of the bacterial 16S rRNA gene (Chen et al., 2018). The PCR system consisted of 5 μL of 10x KOD buffer, 5 μL of 2 mmol/L dNTPs, 2 μL of MgSO_4_, 1 μL of KOD Plus enzyme, 1.5 μL of each primer pair (799F and 1193R), DNA template (total DNA of leaves and endophytes 50 ng), and the total volume was adjusted to 50 μL by adding sterile ddH_2_O. The following PCR conditions were used: initial denaturation at 94°C for 2 min; denaturation at 94°C for 30 s; primer binding at 63°C for 30 s; amplification at 68°C for 30 s, with a total of 30 cycles; final amplification at 68°C for 5 min; and cooling at 4°C indefinitely. PCR for each sample was performed in three biological replicates, and the total amplification product of each sample was the sum of the three replicate PCR products. Finally, high-quality PCR products were further used in library construction and subsequent sequencing analysis using robust Illumina MiSeq™ (PE300).

### 2.4 Sequencing and phylogenetic analysis

To identify operational taxonomic units (OTUs) in sequencing reads, phylogenetic clustering analysis was performed using a Uparse v7.0.10901 OTU clustering tool at a 97% identity threshold level (Ye et al., 2017). Chimeric reads in sequencing data were identified with the help of UCHIME v4.2 software and discarded (Edgar et al., 2011). A group of eight bases was called “word.” The combinations were calculated and compared to the reference library at 100 times the bootstrap cutoff value. The RDP classifier Bayesian algorithm was employed at an 80% confidence threshold level to measure the confidence score of taxa assignment at each taxonomic level. The composition of endophytic bacterial communities in each sample of bamboo leaves was classified into kingdom, phylum, class, order, family, genus, and species (Chen et al., 2016). The common and unique OTU numbers in each sample were counted and illustrated by constructing a Venn diagram (Fouts et al., 2012).

### 2.5 Diversity analysis of endophytic bacteria in bamboo leaves

The diversity of endophytic bacteria in bamboo leaves was analyzed. *α*-Diversity represents sequencing depth, coverage, and comparison of abundance and diversity of endophytes in a microbial community. Good’s coverage metric (C = 1-n1/N) was employed to estimate sequence coverage. Herein, “n1” is the number of OTUs containing only one sequence read, and “N” is the total number of sequence reads obtained from one sample (He et al., 2018). α-Diversity of three biological repeats of each sample was analyzed by employing Chao 1, ACE, Shannon, and Simpson indices (Gihring et al., 2012; Rogers et al., 2016). The linear discriminant analysis (LDA) effect size (LEfSe)^1^ was used to perform the LDA on samples with different grouping conditions according to taxonomic composition. Euclidean distances, dissimilarity indices, and diversity in OTUs at a 97% identity threshold were calculated in each sample by principal coordinate analysis (PCoA) (Calderón et al., 2017). In order to investigate the composition of dominant species in each sample and their distribution among different samples, a Circos sample–species relationship map was constructed with the help of Circos-0.67-7^2^ software (Sui et al., 2016). PICRUSt package was employed for KEGG functional prediction of each 16S amplicon in an OTU (Tang et al., 2018). Comparative taxonomic analyses of the endophytic bacterial community were performed at each classification level in all samples. The R tool was used to construct a community structure diagram and histogram (Lu et al., 2016; Zhou et al., 2017).

### 2.6 Statistical analysis

The analysis was performed using a one-way analysis of variance (ANOVA), with all data points represented as means. Additionally, at a significance level of *p* < 0.05, Tukey’s HSD test was employed to examine differences in means of Shannon, Simpson, ACE, Chao, and diversity indices. All correlation and path coefficient analyses were conducted using Excel 2019 and SPSS Statistics 20.0 (SPSS Inc., Chicago, IL, United States).

## 3 Results

### 3.1 Sequencing data analysis

In triplicate, the 16S rDNA gene was used to analyze the sequence readings of all three bamboo leaf samples, MZ, KZ, and YT (Table 1).

**Table 1:** Sequencing reads analysis of endophytic bacteria in the three samples of bamboo leaves in triplicate.

Sample	Reads	Total bases	Average length (bp)
MZ1	55,924	21,156,612	378.31
MZ2	46,171	17,376,789	376.36
MZ3	55,504	20,965,289	377.73
KZ1	52,248	19,694,141	376.94
KZ2	44,537	16,761,712	376.36
KZ3	47,033	17,699,957	376.33
YT1	51,696	19,475,490	376.73
YT2	57,030	21,458,273	376.26
YT3	48,274	18,169,785	376.39

Column 1 comprises the sample names in triplicate, column 2 comprises the number of sequence reads in each sample, column 3 comprises the total number of bases, and column 4 comprises the average length of sequence reads.

All reads that were incomplete or redundant were eliminated. Full-length recovered reads from MZ bamboo leaf samples were 55,924, 46,171, and 55,504 bp; total number of bases were 21,156,612, 17,376,789, and 20,965,289 bp, and average sequence lengths were 378.31, 376.36, and 377.73 bp. Full-length reads from KZ samples were 52,248, 44,537, and 47,033; the total base count was 19,694,141, 16,761,712, and 17,699,957 bp, and the average read length was 376.94, 376.36, and 376.33 bp. Similarly, in YT samples, the total length sequence reads were 51,696, 57,030, and 48,274, the total number of bases was 19,475,490, 21,458,273, and 18,169,785 bp, and the average sequence length was 376.73, 376.26, and 376.39 bp.

**Figure 1:**
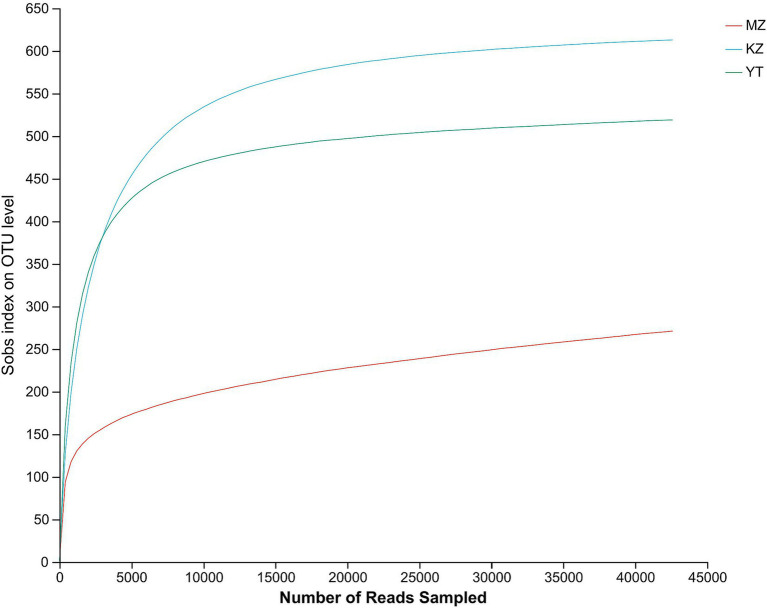
Rarefaction curves of all three samples of bamboo leaves. The abscissa shows the data randomly selected by the Illumina MeSeq™ platform, and the ordinate represents the relative numbers of species. The position of the abscissa of the extension end point of the sample curve represents the number of species in each sample. The flatness of curves indicates a sufficient amount and diversity of bacterial strains, while a steep decline suggests a high proportion of bacterial strains and low bacterial diversity.

The rarefaction curves show the coverage, sampling depth, richness, and uniformity of the endophytic bacterial species present in each bamboo leaf sample. We saw a log phase in all three samples followed by an abrupt flat trend, except for the MZ sample, which had a reasonably short span and a fast decline. The flatness of all three curves demonstrated the abundance of bacterial communities in each sample and sufficient sampling (Figure 1).

### 3.2 OTU cluster analysis

**Figure 2:**
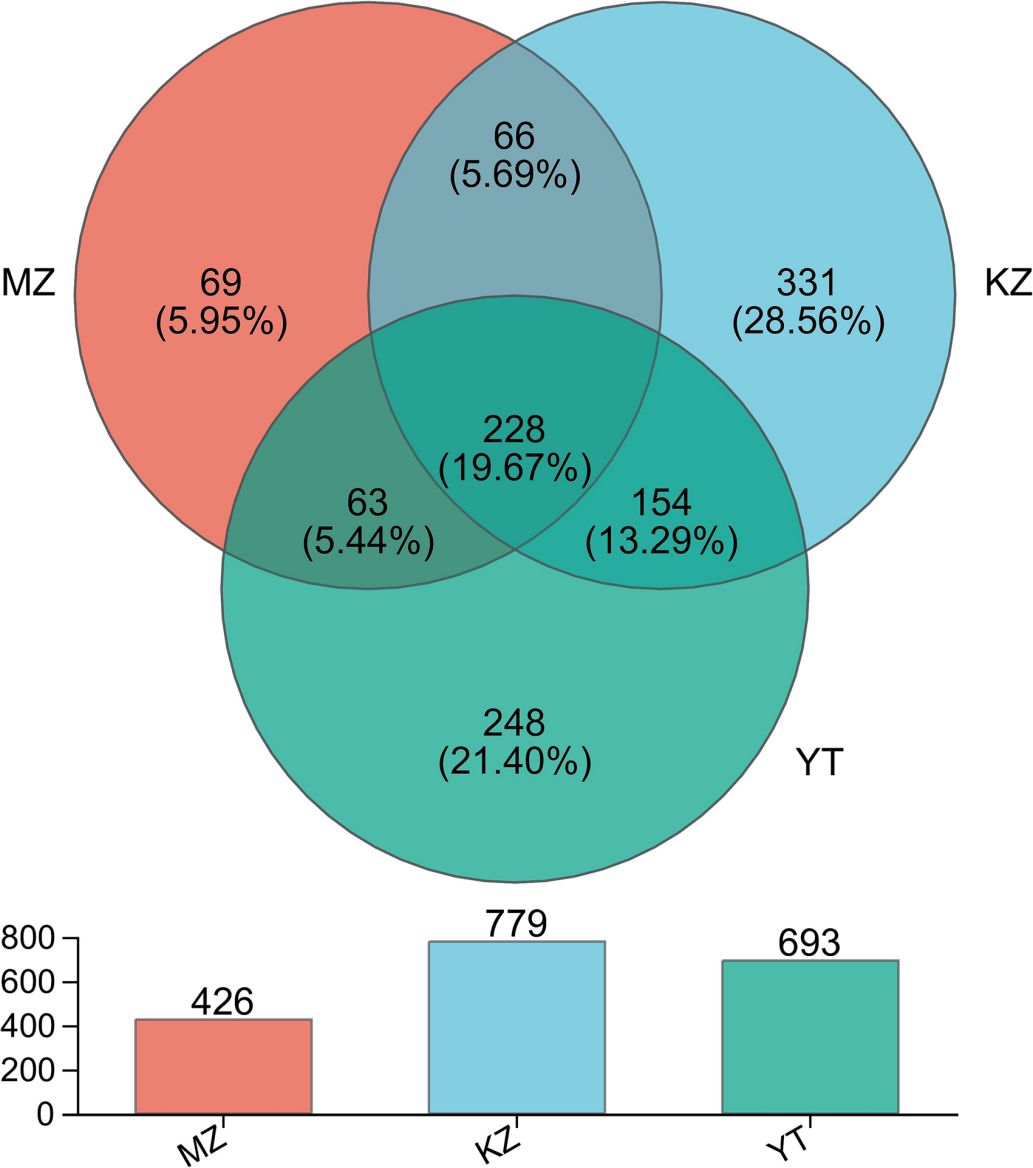
Venn diagram illustrating the unique and common OTUs among all three samples of bamboo leaves. Different colors are assigned to each group. Common and unique OTUs are shown with their numbers. The total number of OTUs in each group, excluding chimeric sequences, is also represented with a bar chart.

In order to illustrate compositional similarity and overlap among OTUs of all three samples, MZ, KZ, and YT, a Venn diagram was constructed (Figure 2).

*In silico* analysis, combined with statistical analysis, revealed 1,159 OTUs via clustering of high-fidelity data free of chimeric reads at a 97% similar index. Furthermore, established OTUs were divided into 26 phyla, 64 classes, 158 orders, 270 families, 521 genera, and 811 species. More precisely, established OTUs in each sample, MZ, KZ, and YT, were 426, 779, and 693, respectively (Figure 2). The Venn diagram exhibited 69 (5.95%) unique OTUs in the MZ sample (peach), 331 (28.56%) in the KZ sample (Tiffany blue), and 248 (21.40%) in the YT sample (green). Furthermore, a total of 66 common OTUs between the MZ and KZ samples (teal), 154 common OTUs between the KZ and YT samples (blue-green), 63 common OTUs between the MZ and YT samples (mint) were observed, and a total of 228 (19.7%) common OTUs were observed among all three samples (Victorian teal). These findings proved that community structures of endophytic bacteria in all three samples of bamboo leaves were significantly different (Figure 2).

### 3.3 Microbial abundance and diversity analysis

The abundance and diversity of endophytic bacterial communities were investigated in bamboo leaves. The average sequencing depths of bamboo leaf samples MZ, KZ, and YT were 0.9990, 0.9986, and 0.998, respectively, demonstrating the real-time coverage of endophytic bacterial communities (Table 2).

**Table 2:** Endophytic bacterial community richness and diversity indices in *Phyllostachys edulis*, *Bambusa rigida*, and *Pleioblastus amarus* leaves.

Sample	Shannon	Simpson	ACE	Chao	Coverage
MZ	2.273 ± 0.132^a^	0.169 ± 0.124^a^	267.792 ± 7.789^a^	285.139 ± 11.234^a^	0.9990
KZ	4.652 ± 0.159^b^	0.042 ± 0.037^b^	617.892 ± 23.942^b^	509.076 ± 13.926^b^	0.9986
YT	5.191 ± 0.219^c^	0.024 ± 0.011^b^	718.705 ± 16.093^c^	543.533 ± 2.501^b^	0.9988

The first column comprises the sample names of bamboo leaves. The second, third, fourth, and fifth columns comprise the Shannon, Simpson, ACE, and Chao indices, respectively. The last column comprises the average values of the diversity indices. Lowercase superscript letters indicate significant differences among samples (*p* < 0.05).

Generally, higher Shannon and lower Simpson indices are indicators of localization of the highest endophytic bacterial diversity index in any biological sample. The highest diversity of the endophytic bacterial community was observed in YT, followed by KZ, and lowest in the MZ group. In order to investigate the richness of communities of bacterial endophytes in bamboo leaf samples, the Chao and ACE indices were also measured. The highest richness of endophytic bacterial communities was observed in YT, followed by KZ, and lowest in the MZ group (Table 2). These results indicate that the richness and diversity of endophytic bacteria in the leaves of all three bamboo species were not adequate.

### 3.4 Bacterial community structure analysis

**Figure 3:**
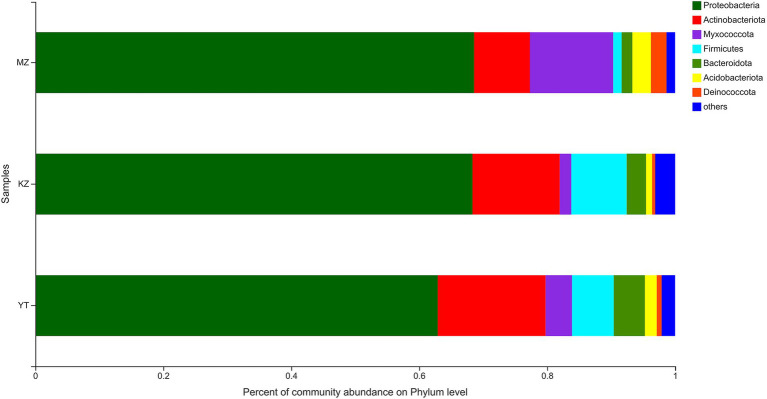
Bar plot analysis of the relative percentage of the endophytic bacterial community at the phylum level. The ordinate represents the names of different samples, and the abscissa represents the relative percentage of different phyla, expressed by columns with different colors and sizes. Phyla with <1% were merged as others.

**Figure 4:**
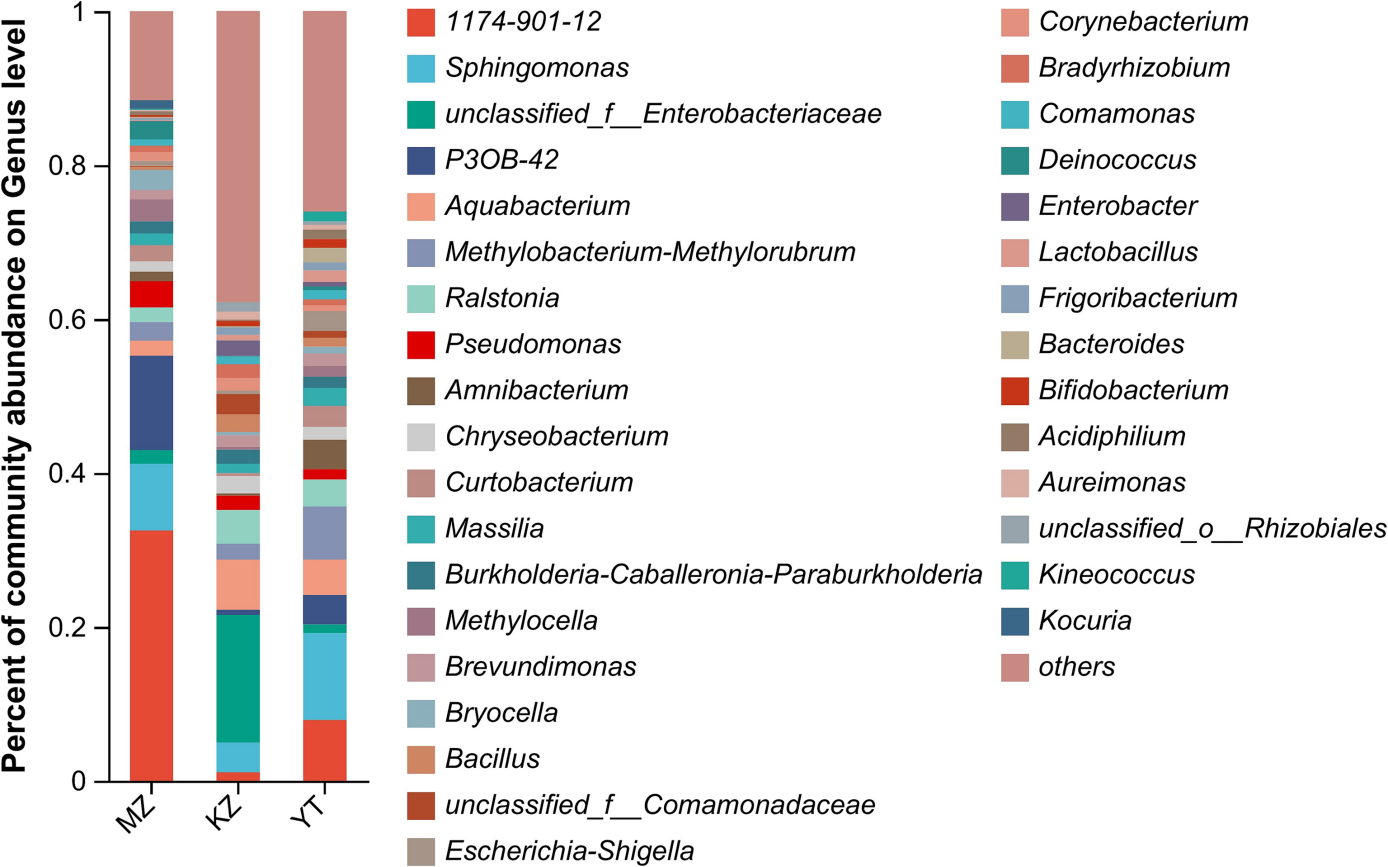
Bar plot analysis of the relative percentage of the endophytic bacterial community at the genus level. The ordinate represents the relative percentage of different genera, and the abscissa represents the names of different samples, expressed by columns with different colors and sizes. Genera with <1% were merged as others.

Relative percentages of community abundance of endophytic bacterium at phylum (Figure 3) and genus levels (Figure 4) have been shown in all three samples of bamboo leaves.

The highly abundant seven phyla were precisely annotated, while <1% relative abundant phyla were merged into “others.” Dominant phyla include Proteobacteria, Actinobacteria, Myxococcota, Firmicutes, Bacteroidota, Acidobacteriota, and Deinococcota. Notably, the proportion of only three phyla, Proteobacteria, Actinobacteria, and Myxococcota, reached more than 90%. The proportion of Proteobacteria was highly abundant in all three samples of bamboo leaves, with MZ, KZ, and YT accounting for 68.58, 68.30, and 62.88%, respectively. Contrarily, the lowest proportion of the Deinococcota phylum was noticed in all three bamboo leaf samples, with MZ, KZ, and YT accounting for 2.42, 0.48, and 0.78%, respectively. The community abundance percentage of Actinobacteria, Firmicutes, and Bacteroidota in the KZ and YT samples was significantly higher than that in MZ. At the same time, Myxococcota and Acidobacteriota were substantially lower in the KZ and YT samples than in the MZ sample (Figure 3).

**Figure 5:**
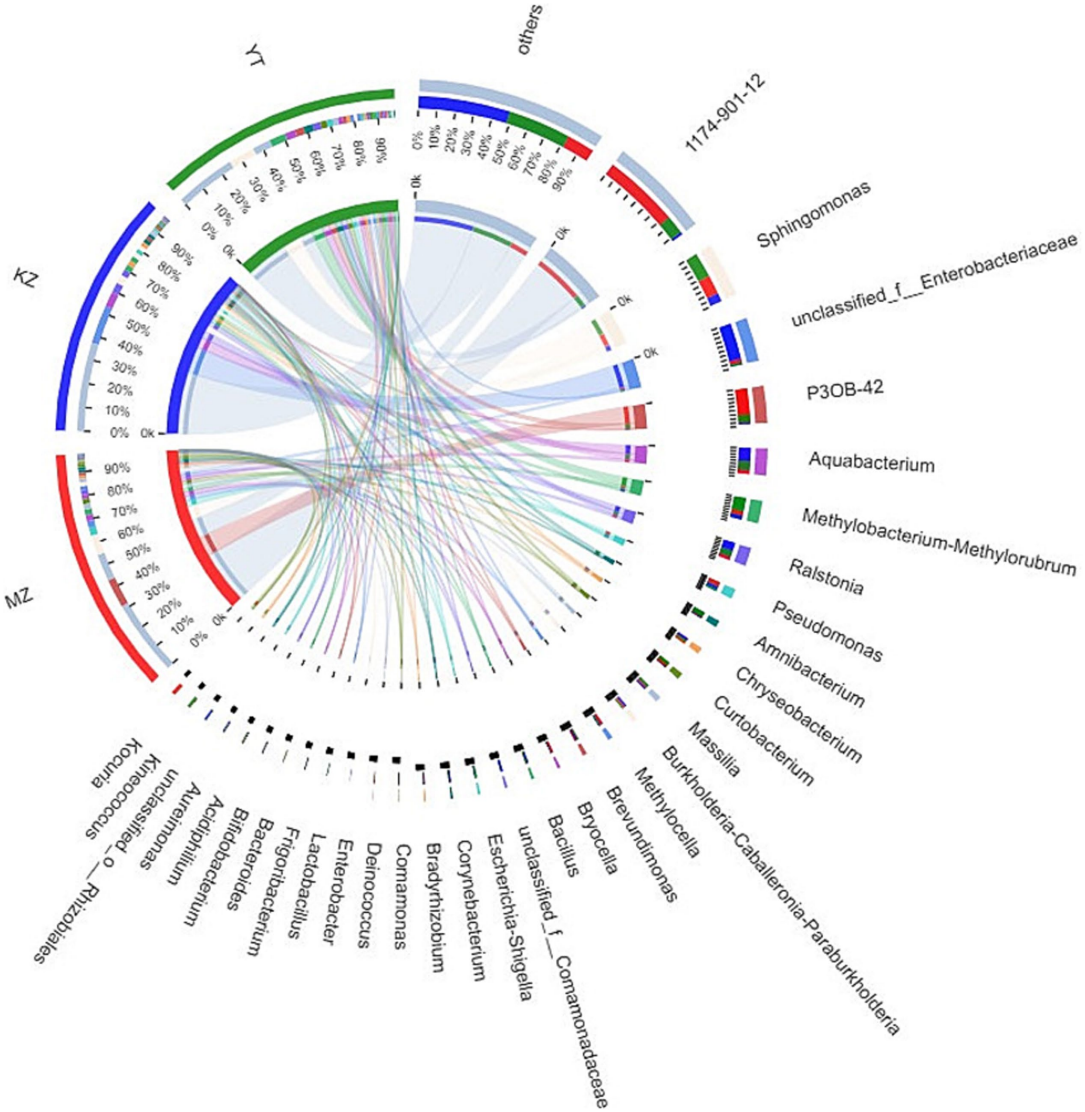
Sample–species relationship-based Circos diagram. The small semicircle (left half circle) represents the composition of species in a sample, the color of the outer ribbon represents different groups, the color of the inner ribbon represents species, and the length represents the relative abundance of the genus in a sample. The large semicircle (right half circle) represents the proportion of genera in different samples.

Notably, an abundance of endophytic bacterial genera was identified in all three samples of bamboo leaves, and genera with abundances <1% were placed under the “others” category (Figure 4). Furthermore, the proportion of dominant endophytic bacterial taxa in each sample and the distribution ratio of each dominant genus across all three samples of bamboo leaves were visually represented using a circular Circos graph (Figure 5). The dominant genera in the MZ sample were 1,174–901-12 (32.54%), *P3OB-42* (12.28%), *Sphingomonas* (8.66%), and *Pseudomonas* (3.39%), while those in the KZ sample were unclassified_f_Enterobacteriaceae (16.58%), *Aquabacterium* (6.51%), *Ralstonia* (4.38%), and *Sphingomonas* (3.87%). In contrast, the composition of the YT sample varied, with *Sphingomonas* (11.29%), 1,174–901-12 (7.89%), *Methylobacterium-Methylorubrum* (6.89%), and *Aquabacterium* (4.58%), although their proportions varied among different bamboo species. Additional research is necessary to determine the precise causes for the fluctuating abundances of various genera in all three bamboo species. In summary, we noted that the abundance of different genera of endophytic bacteria varied among the three bamboo species. The LDA value quantifies species’ impact on the difference effect, while LEfSe assesses multi-level differences between species.

**Figure 6:**
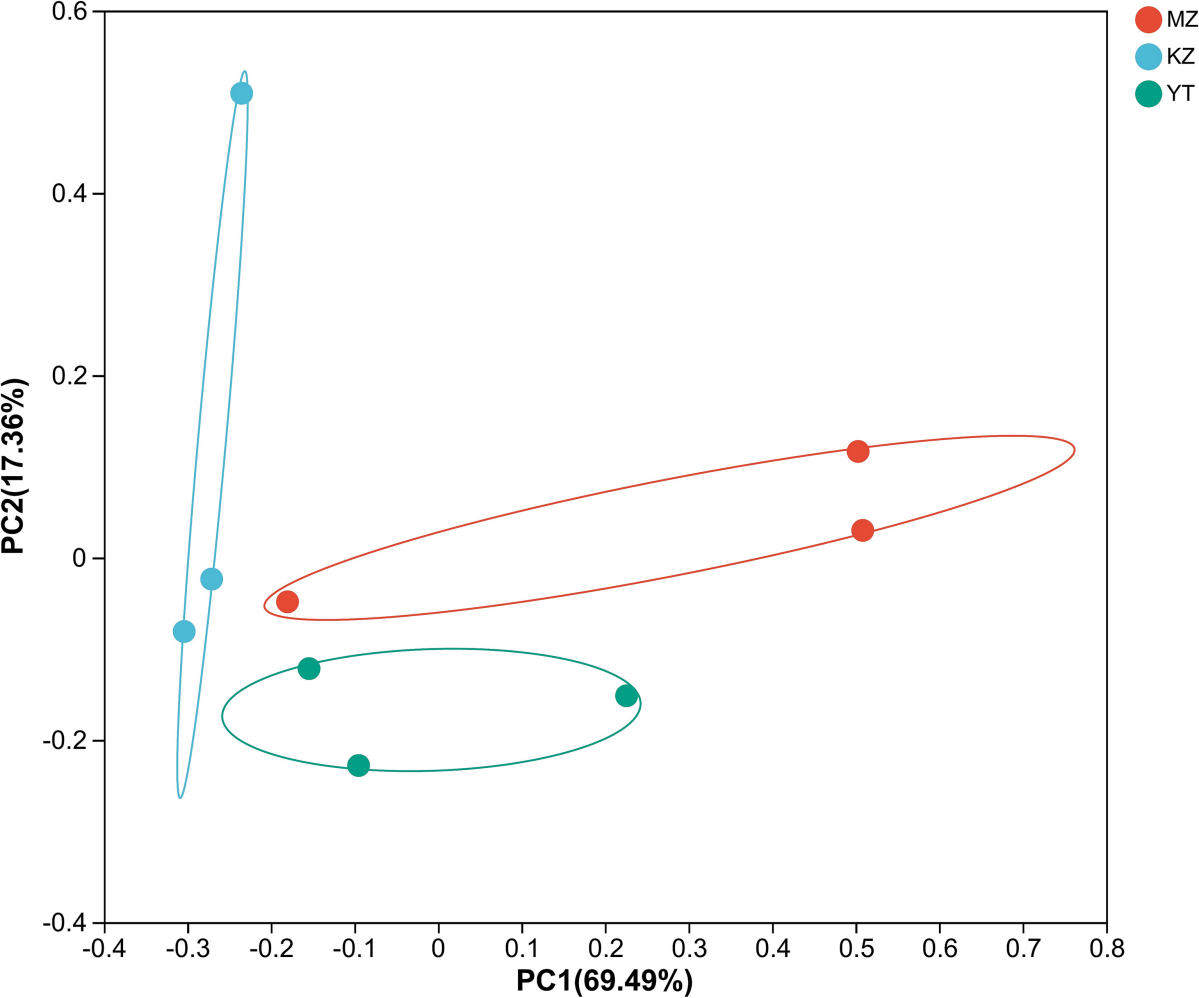
Genus-level discriminant histogram of flora using LDA. The bar graph represents the LDA values for various species. The magnitude of the effect of species abundance on the difference effect increases with the LDA score. In distinct categories, bars of varying colors are used to represent the samples.

This indicates that the aforementioned species potentially significantly influence the environmental change process (Huhe et al., 2017). A bar graph was constructed using the genus with the “LDA value >2,” which represented statistically significant differences among the three bamboo endophytic bacteria as determined by LEfSe analysis (Figure 6). Unclassified_Rhizobiales obtained the highest LDA score in the KZ group, followed by norank_Hyphomicrobiaceae. This suggests that the community structures of the three bamboo endophytic bacteria differed significantly.

### 3.5 Differential analysis of endophytic bacterial communities

**Figure 7:**
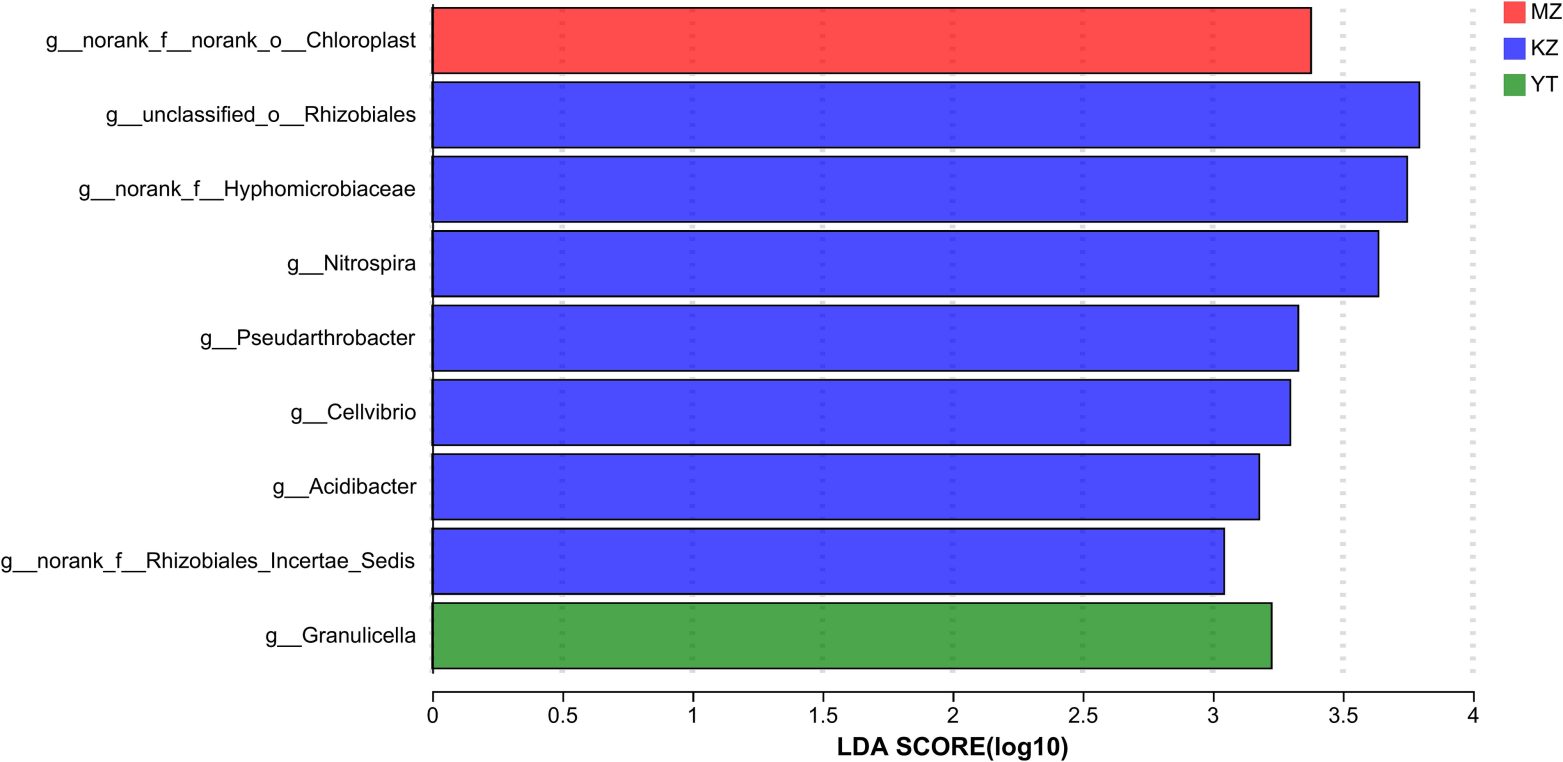
Principal coordinate analysis (PCoA) of multiple samples at the OTU level. The x- and y-axes represent the selected principal component axes, respectively, and the percentage indicates the variation in sample composition by the principal component; the relative distances were denoted on the x- and y-axis scales. In distinct groupings, samples are represented by different color points or shapes. Compositional similarity between bacterial species indicates the proximity of points or structures.

The variation index and Euclidean distances among all three samples were calculated and shown using PCoA (Figure 7).

To create PCoA maps, extraordinarily abundant endophytic bacterial species were identified in each sample. PC1 and PC2 contributed 86.85% to PCoA (Figure 7). The distances between all three bamboo samples were remarkably more significant and dispersed, suggesting that the bacterial population structure was highly diverse.

### 3.6 PICRUSt function prediction analysis

In order to predict functional annotation of Illumina MiSeq™-generated endophytic bacterial 16S amplicons of all three samples of bamboo leaves, PICRUSt software was employed to standardize all OTUs followed by KEGG database, KO, Pathway, and EC information (Table 3).

**Table 3:** Analysis of 16S amplicons using KEGG pathway functions.

Pathway	KZ	YT	MZ
Amino acid metabolism	7,862,424	6,385,023	6,349,137
Biosynthesis of other secondary metabolites	601,828	586,840	597,643
Cancers	113,316	110,651	132,699
Carbohydrate metabolism	8,336,716	6,264,284	6,020,586
Cardiovascular diseases	10,705	15,961	23,093
Cell communication	117	0	3
Cell growth and death	371,006	365,643	449,351
Cell motility	2,699,731	1,951,108	2,258,829
Cellular processes and signaling	3,820,232	2,261,876	2,578,176
Circulatory system	23,506	26,930	30,900
Digestive system	31,294	22,562	20,107
Endocrine system	237,727	242,307	217,628
Energy metabolism	4,080,607	3,228,705	3,698,571
Environmental adaptation	100,894	87,187	98,287
Enzyme families	1,583,703	1,135,584	1,261,019
Excretory system	23,790	22,818	24,781
Folding, sorting, and degradation	1,779,000	1,326,175	1,397,319
Genetic information processing	1,926,739	1,326,722	1,552,598
Glycan biosynthesis and metabolism	1,623,550	1,028,772	1,063,646
Immune system	42,753	28,447	31,685
Immune system diseases	38,602	33,114	30,053
Infectious diseases	431,426	257,005	306,026
Lipid metabolism	2,761,400	2,229,676	2,318,581
Membrane transport	12,208,466	7,485,692	6,806,069
Metabolic diseases	57,601	48,523	54,798
Metabolism	2,426,015	1,510,166	1,587,715
Metabolism of cofactors and vitamins	3,139,831	2,478,433	2,634,511
Metabolism of other amino acids	1,572,640	1,211,369	1,246,408
Metabolism of terpenoids and polyketides	1,513,526	1,282,262	1,303,921
Nervous system	52,740	49,565	37,282
Neurodegenerative diseases	237,899	225,926	259,693
Nucleotide metabolism	2,435,672	1,920,997	1,929,497
Poorly characterized	4,402,210	3,005,545	3,187,329
Replication and repair	5,235,163	4,202,355	4,296,365
Sensory system	39	0	1
Signal transduction	2,110,042	1,425,370	1,561,954
Signaling molecules and interaction	126,400	114,266	128,712
Transcription	2,148,853	1,406,364	1,470,251
Translation	3,023,692	2,449,642	2,545,038
Transport and catabolism	215,949	209,248	199,836
Xenobiotics biodegradation and metabolism	2,685,139	2,214,360	2,038,144

The first column comprises predicted sub-functions in the KEGG pathway, and columns 2–4 comprise abundance values of the KEGG function in each sample.

In total, 41 sub-functions were annotated by PICRUSt software and KEGG database. Functional annotations of all three samples were similar and predominantly involved in metabolic pathways of cellular processes, environmental information processing, genetic information processing, human diseases, metabolism, and organic systems. Dominant sub-functions were relevant to amino acid metabolism, carbohydrate metabolism, cell motility, cellular processes and signaling, energy metabolism, and membrane transport. Significant variations in sub-functional abundances have been observed among all three samples; that is, carbohydrate metabolism was more abundant in KZ than in YT and MZ. Similarly, cell growth and death, cardiovascular diseases, signaling molecules, and interaction were abundant in MZ compared to KZ and YT (Table 3).

## 4 Discussion

Genetic composition, germination, growth time, growth potential, and localization to a specific region primarily influence endophytic bacterial diversity, variable community structure, and abundance in various bamboo species. This study determined that the diversity of endophytic fungi in the leaves of the three bamboo species differed, among which *B. rigida* was the most diverse and abundant endophytic bacteria, followed by *Pl. amarus* and *Ph. edulis*. This can be seen in the disparities between *Populus* varieties concerning endophytic microorganisms’ diversity, as reported by Yuan et al. (2014). Comparable occurrences were noted in the diversity of endophytic fungi across various *Rhodiola* varieties (Cui et al., 2015). High-throughput gene sequencing is a robust and leading technology for precise and quick exploration of the community structure and abundance analysis of endophytic bacteria in a given sample compared to traditional culturing techniques, which are laborious, time-consuming, and even unable to culture numerous endophytes. We observed significant variations in the number of OTUs, the composition of endophytic bacterial communities, and their diversity and richness among different bamboo species. The highest endophytic bacterial community richness was observed in YT and the lowest in MZ, while KZ and YT harbor more OTUs than MZ. These variations could be due to different physiological characteristics and the organizational structure of *B. rigida*, compared to *Ph. edulis* and *Pl. amarus*. Specific tissue structure and more extended localization of *B. rigida* clumps resulted in a higher accumulation of endophytic bacteria. The tissue structure of *B. rigida* (YT) is sympodial, and its genetic makeup is also unique as compared to monopodial and mixed bamboo (Gao et al., 2019), which facilitates the invasion and transmission of microbes (Les, 2020). PCoA revealed that the differences in community structure among the three bamboo endophytic bacteria provided further support for the above-stated hypothesis. The distance was close, and the three samples of *B. rigida* had good repeatability, whereas the *Ph. edulis* and *Pl. amarus* sample*s* were poorly repeatable and relatively dispersed.

Numerous endophytic bacteria are localized inside bamboo leaves, and their genes complement specific biological functions (Suryanarayanan et al., 2022). For example, Proteobacteria, Actinobacteria, and Firmicutes significantly tolerate heavy metal stress and are adaptive to acidic soil, while bamboo also grows well in acidic and heavy metal contaminant soil (Chao et al., 2016; Liu et al., 2021). So, there would be a positive correlation between endophytes and bamboo, which further needs to be investigated. Studies have shown that Proteobacteria positively correlate with soil nutrients and nitrogen metabolism, denitrification under anaerobic conditions in a nitrate-rich environment, and nitrogen fixation in a nitrogen-deficient climate (Gao et al., 2019). Additionally, Proteobacteria can habitat water, sediments, soil, and alkaline and saline environments to support bamboo growth in all habitats (Deng et al., 2020). Actinobacteria produce and secrete secondary metabolites to regulate the carbon cycle of other microbes and promote the growth rate of bamboo plants (Jose et al., 2021). Firmicutes produce spores that can adapt to various environments and play a vital role in development of the bamboo ecosystem (Govil et al., 2021). This study determined the abundance of Proteobacteria, Actinobacteria, and Myxococcota in three kinds of bamboo, indicating an interaction between these bamboo species and endophytes. These endophytes can help bamboo adapt to the local acidic soil environment and humid climate and promote the growth of bamboo and the biosynthesis of secondary metabolites.

Many endophytes have been identified in various bamboo species, and bamboo endophytes are extensively distributed within the host. In their study, Liu et al. (2014) isolated 27 isolates of endophytic bacteria from *Ph. edulis*, the predominant genera of which were *Bacillus*, *Arthrobacter*, *Staphylococcus*, and *Ochrobactrum*. *Bacillus* and *Pseudomonas* were the most prevalent genera of endophytic bacteria in the leaves of six bamboo species, as compared and analyzed by Xu et al. (2014). From *Ph. edulis*, Yuan et al. (2014) isolated 34 endophytic bacteria strains. The prevailing genera were *Arthrobacter*, *Bacillus*, *Curtobacterium*, *Alcaligenes*, and *Staphylococcus*. Forty endophytic bacterial isolates were isolated from *Bambusa blumeana* by Hou et al. (2019), most of which belonged to the taxa *Azospirillum*, *Aquaspirillum*, *Escherichia*, and *Pseudomonas*. However, the community structure of endophytic bacteria in this study differs from that reported in previous studies. The most abundant species identified were *P3OB-42*, *Aquabacterium*, *Methylobacterium-Methylorubrum*, *Sphingomonas*, unclassified_f__Enterobacteriaceae, *1,174–901-12*, *Sphingomonas*, *Ralstonia*, *Pseudomonas*, and *Bacillus*. *Sphingomonas* harbors particular cell membrane-localized components called glycosphingolipids, which can tolerate nutrient-deficient environments and have strong vitality. *Sphingomonas* also degrades macromolecular organic pollutants to control environmental pollution (Li et al., 2021). Enterobacteriaceae are harmless and widely distributed in soil, water, plants, insects, and animals (Ito et al., 2019). *Pseudomonas* is an opportunistic pathogen, and knowledge about its specific role in bamboo leaves is scarce (Williams et al., 2010). *Bacillus* is another widely distributed endophytic bacterium that can be isolated in many plant species. Members of the *Bacillus* genus are mostly probiotics, with numerous beneficial effects in the intestine, fecal frequency and characteristics, and skin properties of animals and humans. For example, *Bacillus coagulans* is a probiotic that can improve the flavor, taste, and shelf life of bamboo-based food (Endres et al., 2011).

In the past, the primary focus of high-throughput sequencing was only to explore the community structure of endophytic bacteria in plants (Ding et al., 2016). In metagenomic research, high-throughput sequencing-based PICRUSt function prediction is highly efficient, convenient, cost-effective, and reliable (Langille et al., 2013). In recent years, functional annotation of various habitats’ microbes has been investigated via PICRUSt (Zhu et al., 2016; Dong et al., 2018). This research predicted key endophytic bacterial species and their roles in biological functions in the leaves of three different bamboo species. The dominant sub-functions were amino acid metabolism, carbohydrate metabolism, cell motility, cellular processes and signaling, energy metabolism, and membrane transport, which are poorly characterized in all three experimental groups. These findings suggest that genetic characteristics, environmental conditions, and community structure are jointly affected by the metabolic functions of endophytes in bamboo. However, due to the limitations of PICRUSt function prediction, combining it with metagenomic sequencing is suggested to understand the microbial community functions of various bamboo species accurately.

Regarding bamboo distribution from the Shunan Bamboo Sea, the three bamboo species have extremely diverse endophytic bacteria, which provides a basis for screening bioactive substances of bamboo endophytic bacteria and developing bamboo resources. The above conclusion may have limitations as microorganisms have a high degree of functional redundancy and cannot accurately predict their functional characteristics. In contrast, functional genes can better predict the response of microbial communities to habitat changes. In the future, more bamboo samples from different regions will be collected for in-depth research using metagenomics methods to reveal the characteristics and diversity of endophytic bacteria in bamboo and their relationship with microbial function.

## 5 Conclusion

Bamboo has numerous ecological, commercial, ornamental, and industrial uses and is the fastest-growing forest resource. Endophytic microorganisms coexist symbiotically with an extensive variety of plant species. The composition of endophytic bacterial populations in the leaves of three different bamboo species was investigated using high-throughput sequencing. We determined that bamboo species have a particular impact on the structure and diversity of endophytic bacterial communities. Endophytic bacterial phyla that were exceptionally abundant included Proteobacteria, Actinobacteria, and Myxococcota. The dominant genera of the MZ sample were *P3OB-42*, *Sphingomonas*, and *Pseudomonas*, while the dominant genera of YT were *Sphingomonas*, 1,174–901-12, and *Methylobacterium*-*Methylorubrum*. *B. rigida* was the most diverse and abundant endophytic bacteria among the three bamboo species, followed by *Pl. amarus* and *Ph. edulis*. According to the PICRUSt function prediction analysis, endophytic bacteria in bamboo leaves are predominately enriched in the six biological pathways: human disease, metabolism, cellular processes, environmental information processing, genetic information processing, and organic systems. This indicates that the metabolic functions of endophytic bacteria in bamboo jointly affect bamboo’s genetic characteristics, environmental conditions, and community structure. In conclusion, the most advantageous microorganisms discovered in this study are beneficial, as they can regulate ecological pollution, perform biological control, and enhance food flavor. These results will guide the cultivation and management of bamboo forests, as well as a reference for the development of efficient and beneficial microorganisms with the potential to solve environmental pollution problems caused by climate change.

## Data Availability

The datasets presented in this study can be found in online repositories. The names of the repository/repositories and accession number(s) can be found in the article/Supplementary material.

## Footnotes

^1^ http://huttenhower.sph.harvard.edu/galaxy/root?tool_id=lefse_upload

^2^ http://circos.ca/
